# Tumor characterization and treatment monitoring of postsurgical human breast specimens using harmonic motion imaging (HMI)

**DOI:** 10.1186/s13058-016-0707-3

**Published:** 2016-05-09

**Authors:** Yang Han, Shutao Wang, Hanina Hibshoosh, Bret Taback, Elisa Konofagou

**Affiliations:** 1 Department of Biomedical Engineering, Columbia University, 351 Engineering Terrace, 1210 Amsterdam Avenue, New York, NY USA; 2 Department of Pathology and Cell Biology, NewYork-Presbyterian Hospital, New York, NY USA; 3 Department of Surgery, NewYork-Presbyterian Hospital, New York, NY USA; 4 Department of Radiology, Columbia University, New York, NY USA

**Keywords:** HMIFU, Human breast tumor, HIFU monitoring

## Abstract

**Background:**

High-intensity focused ultrasound (HIFU) is a noninvasive technique used in the treatment of early-stage breast cancer and benign tumors. To facilitate its translation to the clinic, there is a need for a simple, cost-effective device that can reliably monitor HIFU treatment. We have developed harmonic motion imaging (HMI), which can be used seamlessly in conjunction with HIFU for tumor ablation monitoring, namely harmonic motion imaging for focused ultrasound (HMIFU). The overall objective of this study was to develop an all ultrasound-based system for real-time imaging and ablation monitoring in the human breast in vivo.

**Methods:**

HMI was performed in 36 specimens (19 normal, 15 invasive ductal carcinomas, and 2 fibroadenomas) immediately after surgical removal. The specimens were securely embedded in a tissue-mimicking agar gel matrix and submerged in degassed phosphate-buffered saline to mimic in vivo environment. The HMI setup consisted of a HIFU transducer confocally aligned with an imaging transducer to induce an oscillatory radiation force and estimate the resulting displacement.

**Results:**

3D HMI displacement maps were reconstructed to represent the relative tissue stiffness in 3D. The average peak-to-peak displacement was found to be significantly different (*p* = 0.003) between normal breast tissue and invasive ductal carcinoma. There were also significant differences before and after HMIFU ablation in both the normal (53.84 % decrease) and invasive ductal carcinoma (44.69 % decrease) specimens.

**Conclusions:**

HMI can be used to map and differentiate relative stiffness in postsurgical normal and pathological breast tissues. HMIFU can also successfully monitor thermal ablations in normal and pathological human breast specimens. This HMI technique may lead to a new clinical tool for breast tumor imaging and HIFU treatment monitoring.

## Background

Breast cancer is the most common cancer and the second leading cause of cancer death among women. In the United States in 2013, approximately 300,000 women were diagnosed with breast cancer and almost 40,000 died due to the disease [1]. Image-guided minimally invasive treatment of localized breast tumors has become a subject of intensive research, and researchers in several studies have assessed the feasibility of minimally invasive breast tumor ablation techniques, such as radiofrequency ablation (RFA), cryoablation, and high-intensity focused ultrasound (HIFU) ablation [2]. Minimally invasive treatments apply extreme temperatures, either high or low, to induce irreversible cell injury, tumor apoptosis, and coagulative necrosis [3]. Compared with conventional surgical procedures, the main advantages of minimally invasive ablation lie in the fact that they are less invasive, less scarring, and less painful, allowing for shorter recovery time [4].

HIFU is an entirely noninvasive technique, in which the ultrasound beam is focused on a small target volume to reach high focal power, resulting in temperature elevations causing coagulative necrosis in the target while surrounding structures are spared. Since the ultrasound wave penetrates through soft tissue without any surgical incision or needle insertion, there is no damage to the skin or underlying tissues. Acoustic energy can induce temperature elevations at the focal spot in a few seconds and can simultaneously induce cell death and vascular destruction in normal and tumor tissues [5]. Because of the steep thermal gradients involved, the boundaries of the ultrasound-treated volumes can be closely spaced until the entire target is covered.

Clinically, fibroadenoma (FA) is the most common benign breast mass and may be accompanied by pain [6]. FA does not require treatment; however, palpable FA may cause distress in some patients, who then may request removal. Compared with conventional surgical excision or other minimally invasive techniques, HIFU is completely noninvasive, which provides better cosmetic results and shorter recovery time. Recently, a multicenter study was conducted, in which HIFU was performed as an outpatient procedure in 42 women with 51 FAs [7]. The researchers demonstrated that HIFU is effective in reducing the volume and other clinical symptoms of FA. HIFU has also been studied for ablation of early-stage malignant breast tumors in elderly patients who are not surgery candidates [8–10].

Currently, magnetic resonance imaging (MRI) and sonography are being used for guidance and monitoring of HIFU therapy. Both magnetic resonance-guided high-intensity focused ultrasound (MRgFUS) [11, 12] and ultrasound-guided high-intensity focused ultrasound (USgFUS) methods have their advantages and disadvantages. MRI has the advantage of providing temperature data within seconds after HIFU exposure. However, MRI guidance is expensive and lengthy. Ultrasonic guidance provides the benefit of imaging using the same form of energy that is being used for therapy. Therefore, if the target can be well-visualized with sonography, then the HIFU therapy may avoid potentially causing thermal injury to normal tissue. Worldwide, thousands of patients with uterine fibroids, liver cancer, breast cancer, pancreatic cancer, bone tumors, and renal cancer have been treated with USgFUS [13–15].

Ultrasound elasticity imaging is used in addition to traditional B-mode ultrasound as a tool for differential diagnosis between normal and tumor tissue in the human breast, based on tissue stiffness. Qualitative and quantitative tissue strain analyses using acoustic radiation force impulse (ARFI) imaging technology has been developed for the diagnosis of breast masses [16–18]. The response of tissue to the acoustic radiation force is tracked as tissue displacement, which has been found to be correlated with local stiffness of the tissue. Vibro-acoustic tissue mammography is also a radiation force-based method that uses focused ultrasound to vibrate tissue (in kilohertz), and uses the resulting response to produce images that are related to the hardness of the tissue [19, 20]. Supersonic shear imaging uses a very fast (5000 frames/second) acquisition sequence to capture the propagation of shear waves [21] that provide information on the local viscoelastic properties [22, 23].

Over the past decade, ultrasound elasticity imaging has not been limited to the area of disease diagnosis but has also emerged in the application of therapy guidance and monitoring [24, 25]. Past studies include but are not limited to elastography for HIFU treatment [26–33]. Harmonic motion imaging (HMI) is an all-ultrasound-based elasticity imaging technique designed for both reliable diagnosis and HIFU treatment monitoring. It uses a HIFU transducer to emit an amplitude-modulated (AM) beam for thermal therapy while inducing a stable oscillatory tissue displacement at its focal zone. The oscillatory response, namely HMI displacement, is estimated using the radiofrequency (RF) signals recorded during the HIFU treatment through a confocally aligned imaging transducer [34, 35]. The localized tissue response is monitored continuously from the onset of HIFU treatment and is aimed at providing clinicians the change in local tissue stiffness to prevent any under- or overtreatment. Since HMI does not interrupt HIFU ablation, HIFU sonication was operated with a duty cycle of 100 %. Several studies have been published as feasibilities ex vivo [36, 37], and in vivo [38, 39] using 1D [40] and 2D [41] systems. The overall goal of this study was to develop and test an HMI system for real-time imaging and ablation monitoring in postsurgical breast specimens.

## Methods

**Fig. 1:**
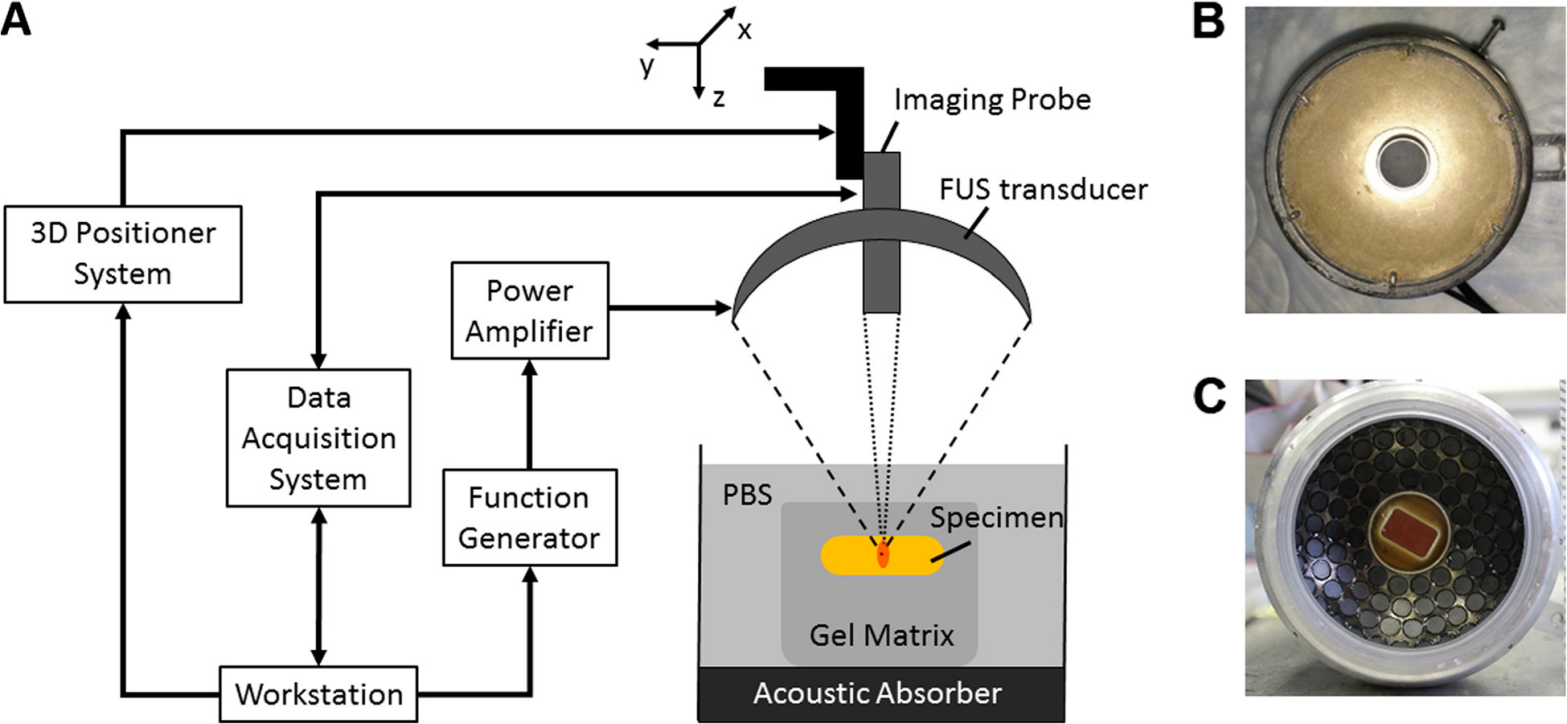
Schematic of harmonic motion imaging (HMI) system with experiment setup. **a** Block diagram of HMI system. The *red* region indicates the focus of the focused ultrasound (FUS) transducer. **b** The 1D HMI system comprised of a single-element FUS transducer (outer diameter 80 mm, inner diameter 16.5 mm) coaligned with a single-element pulse-echo transducer (diameter 15 mm). **c** The 2D HMI system consisted of a 93-element FUS phase array transducer (outer diameter 110 mm, inner diameter 41 mm) and a 64-element phase array imaging probe

Collection and handling of postsurgical breast specimens were approved by the institutional review board of Columbia University, and informed consent was obtained from all enrolled patients. Thirty-eight tissue specimens were obtained from 24 patients who underwent lumpectomy or mastectomy, including 19 normal, 15 invasive ductal carcinomas (IDCs), and 2 FAs. The age range of the patients was 21–89 years (mean age 60 ± 18 years). All specimens were immediately immersed in PBS after surgery and transported in ice. The specimens used for the experiments were approximately 2 cm × 2 cm × 1 cm in size and were received from the Department of Pathology at Columbia University Medical Center. Specimens were embedded in agar gel matrix soaked in degassed PBS in a water tank, as shown in Fig. 1. The tank wall was lined with a layer of sound-absorbing material to reduce undesired echoes from below.

### HMI

HMI measures the vibrational response of the target region to an oscillatory radiation force. The radiation force is caused by the change in momentum of the acoustic wave as it propagates through a medium. In an attenuating homogeneous medium, and assuming plane wave propagation, this force can be expressed as [42, 43]:

1 $$ F=\frac{2\alpha I}{c} $$

where *F* is a volumic force (N/m^3^), *α* is the tissue absorption coefficient (m^−1^), *I* is the temporal average acoustic intensity (W/m^2^), and *c* is the speed of sound [m/second]. When an AM waveform is used to drive the focused ultrasound (FUS) transducer, the radiation force is oscillating at the modulation frequency *ω*_*m*_, which is 25 Hz in this study. In HMI, an oscillatory response is induced at the HIFU focal zone due to the AM-HIFU excitation, namely the HMI displacement. This HMI displacement can be monitored throughout the entire HIFU treatment duration. The change of the peak-to-peak HMI displacement amplitude can be correlated with the relative change in local tissue stiffness as the thermal lesion develops.

A schematic illustration of the main components of the HMI system is shown in Fig. 1. HMI was performed using both 1D and 2D HMI systems in this study. The 1D HMI system was comprised of a single-element FUS transducer (outer diameter 80 mm, inner diameter 16.5 mm, center frequency 4.75 MHz, focal depth 90 mm; Riverside Research Institute, New York, NY, USA) coaligned with a single-element pulse-echo transducer (diameter 15 mm, center frequency 7.5 MHz, focal depth 60 mm; Olympus-NDT, Waltham, MA, USA) (Fig. 1). The FUS transducer was driven by an amplitude-modulated (AM frequency *ω*_*m*_ 25 Hz) sinusoidal signal generated by a dual-channel arbitrary waveform generator (AT33522A; Agilent Technologies Inc., Santa Clara, CA, USA) and amplified by a nominal 50-dB gain power amplifier (325LA; Electronics & Innovation [E&I], Rochester, NY, USA). The pulse-echo transducer was mounted through the center hole of the FUS transducer and confocally aligned with it. It was connected to a pulser-receiver (Olympus-NDT) operating at 1 kHz. The received RF signals from the pulser-receiver were band-pass filtered (Reactel Inc., Gaithersburg, MD, USA) with cutoff frequencies of f_c1_ = 5.84 MHz and f_c2_ = 8.66 MHz and then recorded with a digitizer (GaGe; DynamicSignals, Lockport, IL, USA) at a sampling frequency of 100 MHz. To generate a 3D HMI displacement map, point-by-point raster scan acquisition was used with a step size of 0.5 mm in transverse plane. At each spot, the FUS exposure was 0.6 seconds long (30-cycle oscillations at 50 Hz), during which 600 RF lines at 1-kHz pulse repetition frequency were acquired.

The 2D HMI system consisted of a 93-element FUS phased array transducer (individual element diameter 10 mm, overall outer diameter 110 mm, inner diameter 41 mm, center frequency 4.5 MHz, focal depth 70 mm, H-178; Sonic Concepts Inc., Bothell, WA, USA) and a 64-element phase array imaging probe (center frequency 2.5 MHz, P4-2; ATL Ultrasound, Bothell, WA, USA) (Fig. 1). In this feasibility study, the 93-element FUS transducer was driven in phase using the same function generator and power amplifier as the 1D system. The imaging probe was inserted through an opening in the center of the FUS transducer and confocally aligned with it. The imaging probe was operated with an ultrasound imaging research system (V-1; Verasonics, Kirkland, WA, USA). The FUS total output acoustic power was 11 W in the 1D system and 8.7 W in the 2D system from radiation force balance measurement [44].

### Harmonic motion imaging for focused ultrasound

Harmonic motion imaging for focused ultrasound (HMIFU) ablation monitoring was performed in 19 normal, 15 IDC, and 2 FA specimens using either a 1D or 2D HMI system, and each specimen was ablated at one or two different targeted locations, depending on the size of the specimen. Before HIFU ablation, a standard B-mode image of the targeted region was acquired using the imaging probe. Then HIFU was applied for a total duration of 120 seconds in a single location. Since HMIFU does not require interruption of HIFU ablation, HIFU sonication was operated with a duty cycle of 100 %. A customized plane wave imaging sequence was developed using the Verasonics Data Acquisition System platform. A GPU-based sparse-matrix algorithm was used for fast beamforming. During HMIFU exposure, 200 beamformed frames at a frame rate of 1 kHz were acquired and transferred to the host workstation every 3 seconds. An interpolation was performed to upsample the RF signals by a factor of 8 to achieve a sampling frequency of 80 MHz before storing the RF signals on the host workstation. After 120 seconds of continuous HIFU ablation, a total of 40 datasets were acquired. The main parameters of the two systems are summarized in Table 1.

**Table 1:** Main parameters of 1D and 2D HMI/HMIFU systems

	1D HMI/HMIFU	2D HMI/HMIFU
FUS transducer	4.75-MHz single-element transducer	4.5-MHz 93-element FUS phased array
Imaging transducer	7.5-MHz single-element pulse-echo transducer	2.5-MHz 64-element phased array
Parameters		
AM frequency	25 Hz	25 Hz
Sampling frequency	100 MHz	80 MHz
Frame rate	1000 Hz	1000 Hz
Acoustic power	11 W	8.7 W
Imaging duration	0.6 seconds	0.6 seconds
Ablation duration	120 seconds	120 seconds

*AM* amplitude-modulated, *FUS* focused ultrasound, *HMI* harmonic motion imaging, *HMIFU* harmonic motion imaging for focused ultrasound

### HMI displacement estimation

The signal processing techniques were the same for the 1D and 2D HMI systems. Each set of continuously acquired RF lines using the 1D HMI system, or each set of the 200 continuously acquired RF frames using the 2D HMI system, was processed together. The interference of the FUS beam with the RF signals was removed by digital low-pass filtering (f_cutoff_ = 4 MHz) during processing using the 2D system. In the 1D system, the interference of the FUS beam was removed by the analog band-pass filter as described previously. The incremental axial tissue displacements were estimated by performing a fast 1D normalized cross-correlation between sequentially acquired tracking lines [45]. The RF window size was equal to 5 wavelengths of the imaging probe, and the window overlap was 95 %. A threshold (*R* ^2^ > 0.7) was applied to eliminate poor displacement estimation. The mean peak-to-peak HMI displacement amplitudes and standard deviation of the 10-cycle oscillations at 50 Hz were calculated based on the 200 RF lines or frames. The aforementioned processing was repeated at each raster scan point to obtain the 3D HMI displacement image for breast tumor detection.

For HMIFU ablation monitoring, the mean and standard deviation of the peak-to-peak amplitude of the estimated HMI displacements within the region of interest (ROI) were analyzed for the whole HMIFU ablation process. To compare different targeted locations, normalization of the HMI displacements by the displacement obtained at *t* = 2 seconds was performed. Changes in the HMI displacement amplitude were calculated by the percentage change in HMI displacement at the end of the thermal treatment over the displacement at *t* = 2 seconds.

A focal spot localization method [37] was used to define the ROI. A maximum peak-to-peak displacement value was estimated along the axial direction (1D) or the image (2D). On the basis of the maximum peak-to-peak displacement value, we calculated a −3 dB displacement focal zone over the axial direction (1D) or the image (2D). To quantitatively analyze the relative tissue stiffness change during heating, the resulting displacement around the focal region was then averaged within the −3 dB region.

**Fig. 2:**
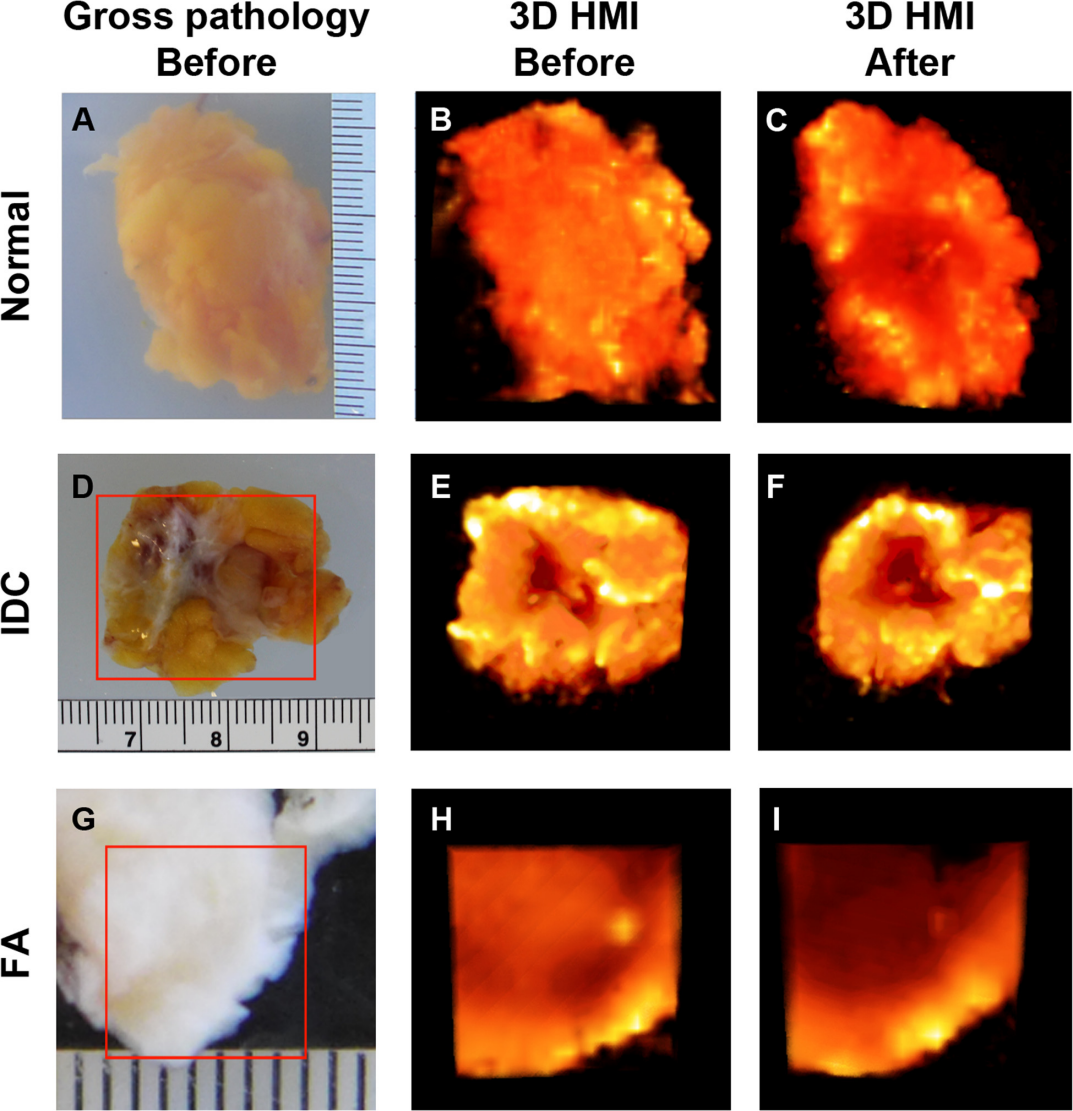
3D harmonic motion imaging (HMI) displacement images of normal breast, invasive ductal carcinoma (IDC), and fibroadenoma (FA) tissue. **a** Gross pathology photograph of a normal breast specimen mounted on the gel matrix. The 3D reconstructed HMI of the selected tissue is shown (**b**) before and (**c**) after harmonic motion imaging for focused ultrasound (HMIFU) ablation. **d** Gross pathology photograph of a IDC specimen mounted on the gel matrix. The 3D reconstructed HMI of the selected tissue is shown (**e**) before and (**f**) after HMIFU ablation. **g** Gross pathology photograph of an FA specimen mounted on the gel matrix. The 3D reconstructed HMI of the selected tissue is shown (**h**) before and (**i**) after HMIFU ablation. The brighter the color, the higher the HMI displacement and the lower relative stiffness, and vice versa

3D data rendering (Fig. 2b, d, f) was achieved using Amira software (FEI, Hillsboro, OR, USA). MATLAB software (MathWorks, Natick, MA, USA) was used for the statistical analysis. The results were presented in mean ± standard deviation format. A paired Student’s *t* test was used to determine a significant difference (*p* < 0.05) between before and after ablation.

### Histology

The specimens were kept in 10 % phosphate-buffered formalin at low temperature (4 °C) for at least 24 h. The specimens were then processed and embedded in paraffin, cut into 4-μm-thick slices, and stained with hematoxylin and eosin (H&E).

## Results

### HMI

**Fig. 3:**
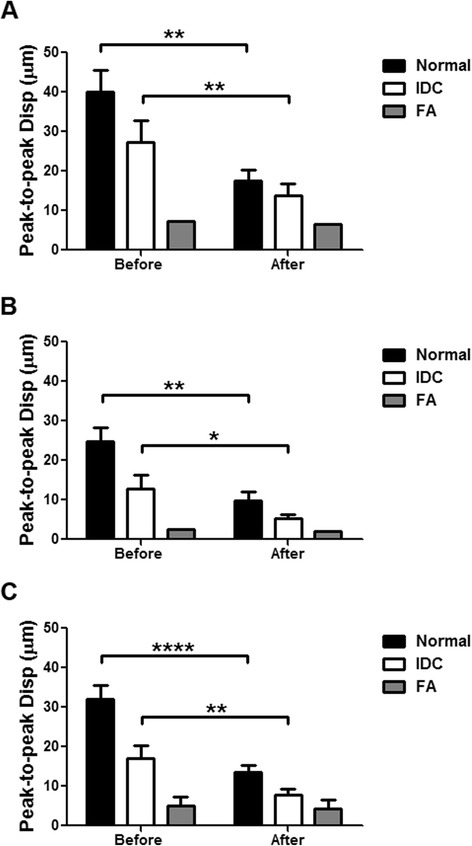
Harmonic motion imaging (HMI) displacement change between before and after ablation. **a** Nine normal, five invasive ductal carcinoma (IDC), and one fibroadenoma (FA) specimens were imaged with the 1D HMI system. **b** Ten normal, ten IDC, and one FA specimens were imaged with the 2D HMI system. **c** Combined results with both HMI systems. *p < 0.05, **p < 0.001, and ****p < 0.00001

In Fig. 2, 3D HMI images of a normal breast specimen, a breast tumor specimen, and an FA specimen before and after HMIFU ablation are shown in comparison with gross pathology images. In the 1D system, the HMI displacement amplitudes of the normal breast, IDC, and FA tissue averaged within the −3 dB regions were 40.10 ± 15.50 μm (*n* = 9), 24.90 ± 9.64 μm (*n* = 5), and 7.40 μm (*n* = 1), respectively (Fig. 3a). With the 2D system, the corresponding values were 24.73 ± 10.97 μm (*n* = 10), 12.77 ± 10.30 μm (*n* = 10), and 2.56 μm (*n* = 1) (Fig. 3b). In histological analysis, we found no tissue damage in the normal breast, IDC, or FA tissue on the basis of HMI imaging alone.

### HMIFU ablation monitoring

Figure 3 shows HMI displacement change before and after HIFU ablation using the 1D (Fig. 3a) and 2D (Fig. 3b) systems. Tissue motion during heating is visible through the periodic variation in displacement amplitude, which is denoted by alternating red (positive) and blue (negative) displacement in Fig. 3. Red denotes the highest motion towards the transducer, and blue represents the highest motion away from the transducer. Using the 1D HMI system, nine (100 %) out of nine normal breast lesions, five (100 %) out of five IDC lesions, and one (100 %) out of one FA lesions were found to have lower HMI displacement amplitude after HMIFU treatment, indicating protein denaturation and necrosis. Using the 2D HMI system, nine (90 %) out of ten normal breast lesions, eight (80 %) out of ten IDC lesions, and one (100 %) out of one FA lesions were found to have lower HMI displacement after HMIFU treatment. HMI displacement before and after HMIFU ablation were compared by performing a paired Student’s *t* test. The mean HMI displacement before and after ablation is shown in Table 2.

**Table 2:** Harmonic motion imaging displacement before and after harmonic motion imaging for focused ultrasound ablation

	1D system				2D system			
	Displacement (μm)				Displacement (μm)			
	Before	After	*n*	*p* value	Before	After	*n*	*p* value
Normal	40.10 ± 15.50	17.49 ± 7.85	9	0.0025	24.73 ± 10.97	9.83 ± 6.46	10	0.0048
IDC	24.90 ± 9.64	12.28 ± 5.44	5	0.0068	12.77 ± 3.50	5.35 ± 2.74	10	0.045
FA	7.34	6.58	1		2.56	2.06	1	

*FA* fibroadenoma, *IDC* invasive ductal carcinoma

**Fig. 4:**
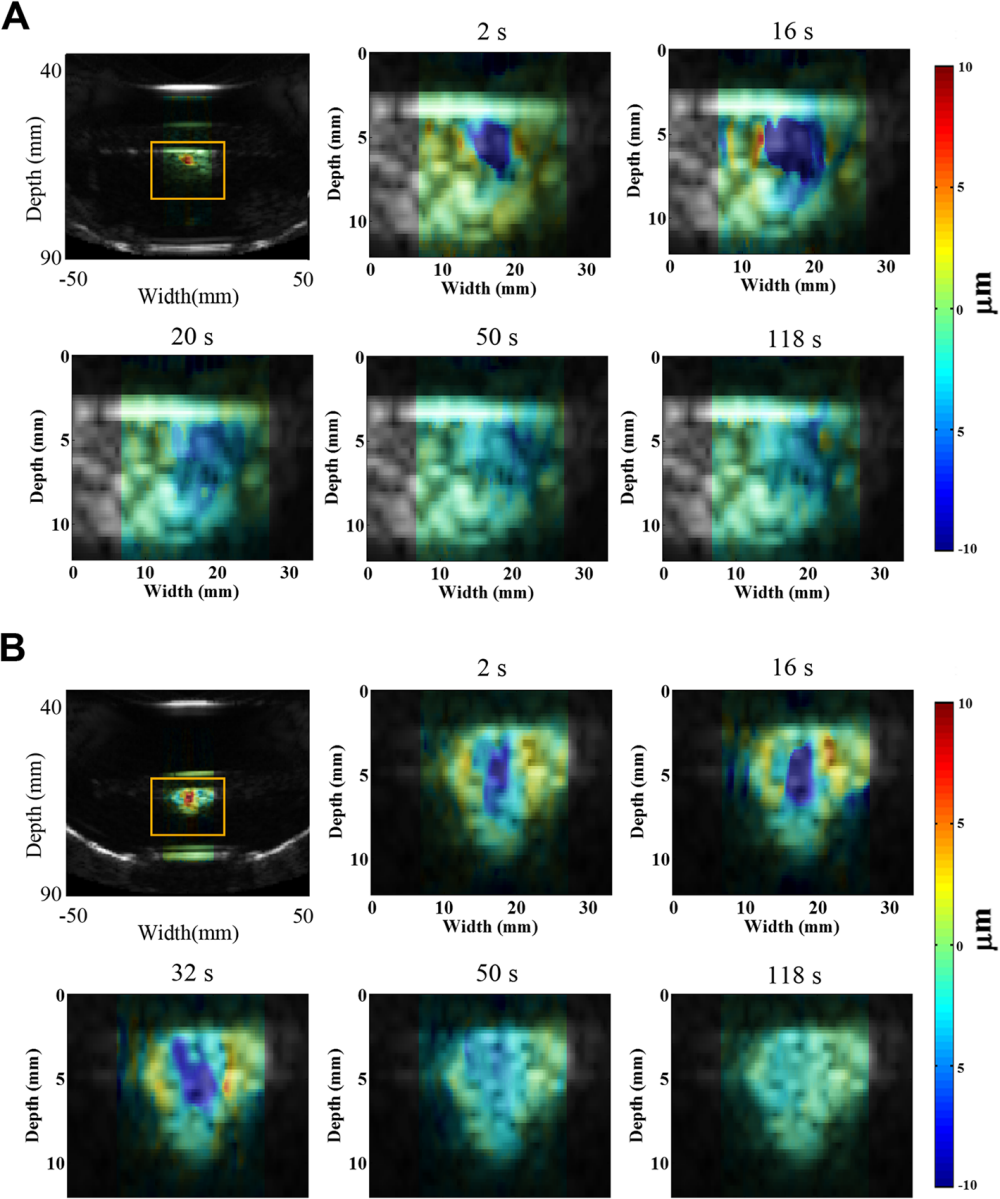
Harmonic motion imaging for focused ultrasound (HMIFU) ablation monitoring in 2D overlaying on B-mode images in (**a**) normal breast and (**b**) IDC tissue. Tissue motion during heating is denoted by alternating *red* and *blue. Red* represents the motion moving toward the transducer, and *blue* represents the motion moving away from the transducer. Peak negative harmonic motion imaging displacement frames during a 50-Hz cycle at five representative timepoints were selected from the HMIFU treatment monitoring sequence to show the decrease of focal displacement as the thermal lesion forms

The mean HMI displacement of the normal breast tissue decreased from 40.10 ± 15.50 μm to 17.49 ± 7.85 μm (*n* = 9, *p* = 0.0025) using the 1D system and from 24.73 ± 10.97 μm to 9.83 ± 6.46 μm (*n* = 10, *p* = 0.0048) using the 2D system. The mean HMI displacement of IDC decreased from 24.90 ± 9.64 μm to 12.28 ± 5.44 μm (*n* = 5, *p* = 0.0068) using the 1D system and from 12.77 ± 3.50 μm to 5.35 ± 2.74 μm (*n* = 10, *p* = 0.045) using the 2D system. The mean HMI displacement of FA decreased from 7.34 μm to 6.58 μm (*n* = 1) using the 1D system and from 2.56 μm to 2.06 μm (*n* = 1) using the 2D system. A similar decrease was also clearly observed in 2D, where the individual frameset consisted of peak negative displacement profiles (Fig. 4) as an example of a treated location.

**Fig. 5:**
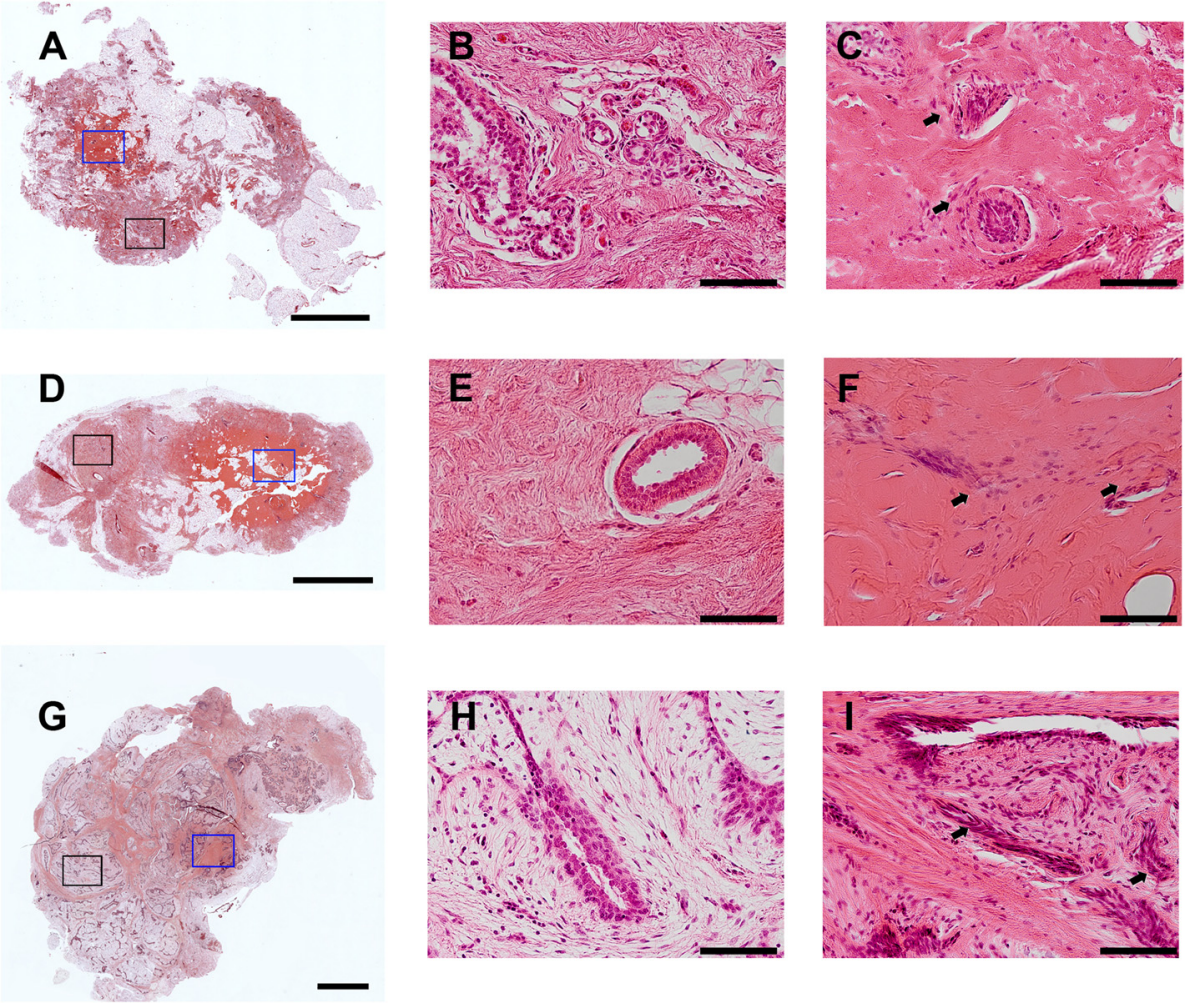
Examples of hematoxylin and eosin staining of harmonic motion imaging for focused ultrasound-ablated normal breast (**a**–**c**), invasive ductal carcinoma (**d**–**f**), and fibroadenoma (**g**–**i**) tissues. In (**a**), (**d**), and (**g**), scale bars indicate 4 mm; in the rest of the images, scale bars indicate 0.1 mm. In (**b**), (**e**), and (**h**), high-magnification images display unablated regions taken within the corresponding *black* frames. In (**c**), (**f**), and (**i**) high-magnification images display ablated regions taken within the corresponding *blue* frames. The ablated regions show discrete hypereosinophilic areas reflecting changes occurring in the collagenous stroma as well as cautery-like “streaming” phenomena (*arrows*) in the HIFU-ablated epithelial regions. The changes seen in the epithelium and/or nuclei are (nonneoplastic, benign, or malignant) reminiscent of what is seen in tissue exposed to electrocautery change. Surrounding tissues remained histologically intact

The normalized HMI displacement change over the entire HMIFU ablation process in all specimens is shown in Table 3. Compared with the initial displacement at the beginning (*t* = 2 seconds), HMI displacement at the end (*t* = 118 seconds) of the HIFU ablation was observed. The HMI displacement amplitudes in normal breast and IDC tissue were 53.84 % and 44.69 % lower, respectively, at the end of the 2-minute HMIFU treatment compared with those at the beginning of HMIFU exposure, showing consistent stiffening after HMIFU ablation. The representative H&E staining results after HMIFU are shown in Fig. 5. Characteristic and similar histologic changes are seen in normal breast parenchyma, FA, or IDC (i.e., all tissues exposed to HIFU). The sections showed discrete hypereosinophilic areas reflecting changes occurring in the collagenous stroma as well as cautery-like “streaming” phenomena in the HIFU-ablated epithelial regions. The changes seen in the epithelium and/or nuclei were (nonneoplastic, benign, or malignant) reminiscent of what is seen in tissue exposed to electrocautery change. The surrounding tissues were histologically unchanged.

**Table 3:** Normalized harmonic motion imaging displacement change over the entire ablation process

	Percentage change	SD
Normal	−53.84 %	25.60 %
IDC	−44.69 %	29.55 %
FA	−15.33 %	4.26 %

*FA* fibroadenoma, *IDC* invasive ductal carcinoma

## Discussion

The clinical application of HIFU in tumor treatment is currently hampered by the lack of a simple, cost-effective device to reliably monitor HIFU treatment that can be used at the point of care. Experimental HMI images presented in this study demonstrate two important facts: (1) HMI is capable of detecting and imaging breast tumors in situ, and (2) HMIFU provides the capability of monitoring tissue stiffness changes while simultaneously probing and forming lesions within the breast tissue.

It was found that HMI displacement amplitudes in the IDC and FA were, on average, 43 % and 85 % lower, respectively, than that in the normal breast. Although the tissue elasticity cannot be directly measured, this displacement contrast ratio can be indexed for relative stiffness of different types of tissues in the breast, considering that a similar level of radiation force was applied. Histological analysis shows that, in the setting of low FUS exposure during tumor detection, no tissue damage is found, which demonstrates that HMI can be performed without causing tissue damage.

Besides tumor detection, HMI offers unique solutions for HIFU tumor ablation monitoring. In this study, for the thermal treatment of tumors ex vivo, an acoustic power of 11 W or 8.7 W and a duration of exposure of 120 seconds for each location were used. Significant reduction (53.84 % decrease in normal tissue and 44.69 % decrease in IDC tissue) in the HMI displacement amplitude in the tumor after HIFU ablation was observed. This indicates that the HIFU-induced lesions could be detected through the reduction in the harmonic motion amplitude. HMI monitors lesion formation based on the underlying tissue stiffness changes. Therefore, it can be more reliable than monitoring lesion formation by tracking the presence of cavitation or boiling bubbles, which are by definition stochastic phenomena currently used in the clinic for HIFU treatment monitoring [46]. We demonstrated that HMI is feasible for monitoring HIFU ablation in human breast tissue without interrupting HIFU treatment.

Application of HMI for breast imaging and HIFU monitoring is both promising and challenging. The overall displacement acquired with the 1D system was higher than that acquired with the 2D system due to the acoustic output difference between the two systems. It may be due to the higher efficiency of the 1D system, which generated higher radiation force and therefore higher displacement in the focal region. The output acoustic power levels used in the two systems were 11 W and 8.7 W, respectively. Both systems, however, showed similar displacement contrast between normal breast, IDC, and FA tissues after normalization. Therefore, the power difference in this study does not influence the ability of HMI to differentiate abnormal from normal tissues. However, it should be noted that the very low acoustic power output could potentially reduce the signal-to-noise ratio with depth. Another limitation of this study lies in the raster scanning used in the experiment for 3D HMI. It involves a relatively lengthy process and thus is subject to motion artifacts induced by respiratory and cardiac motions when applied in patients. Electronic steering of the FUS beam is being used in ongoing studies to sweep the focal spot in order to facilitate the imaging process. Furthermore, although offline processing was used in this study, the 2D HMI system has the fundamental capability of online monitoring [41]. Ongoing work explores the potential of using HMI to provide feedback control of the HMIFU ablation to control HMIFU therapy.

Last, 2D HMI clearly located the HMIFU focal region and monitored the stiffness change of the focal region over time. However, because of the difficulty involved in sectioning through the excised breast to coregister with the imaging plane of the HMI, precise quantitative comparisons of actual lesion size and HMI displacement images cannot be performed with high confidence. To assess the thermal lesion size on the basis of HMI images, additional studies are needed to correlate the HMI displacement map with histological measurements and to define a quantitative threshold in HMIFU displacement change that corresponds to lesion formation, allowing physicians to easily identify the onset of lesion formation. The correlation between tissue structures and HMI characteristics will also be considered in future studies to improve understanding of tissue mechanical responses during HMIFU ablation.

HIFU has been available in the United States to treat uterine fibroids and prostate tumors and to relieve pain due to bone metastases. Moreover, there is a growing number of clinical applications at various stages of research and development around the world, including breast cancer [47], pancreatic cancer [48], and brain tumors [49]. MRgFUS has the advantage of high spatial resolution with thermometry capability. However, the intrinsic low frame rate of MRI prohibits real-time monitoring of the treatment. On the other hand, USgFUS allows for real-time imaging at a relatively low cost. Different from conventional B-mode monitoring, which relies on the appearance of hyperechoic spots resulting from boiling, HMIFU can provide relative tissue stiffness changes in real-time without interfering with the HIFU treatment. Future clinical HMIFU could thus be used in an outpatient setting. In addition to its relatively low associated costs, HMIFU will also be characterized by its noninvasive nature and reduced adverse effects.

The present study lays the foundation for future development of HMI as a clinical monitoring technique for breast HIFU with the added capability of detecting tumors for treatment planning, evaluation of tissue stiffness changes during HIFU ablation for treatment monitoring in real-time, and assessment of thermal lesion sizes after treatment evaluation.

## Conclusion

In this paper, we present a noninvasive imaging method for imaging postsurgical breast tumor specimens and monitoring of HIFU ablation. The results of this study indicate that breast tumors confirmed with pathology can be detected by HMI. HMI has been shown experimentally to be capable of mapping and differentiating stiffness in normal and abnormal breast tissues. HMIFU can also successfully generate thermal lesions on normal and pathological breast tissues.

## Abbreviations

AM: amplitude-modulated
ARFI: acoustic radiation force impulse
FA: fibroadenoma
FUS: focused ultrasound
H&E: hematoxylin and eosin
HIFU: high-intensity focused ultrasound
HMI: harmonic motion imaging
HMIFU: harmonic motion imaging for focused ultrasound
IDC: invasive ductal carcinoma
MRgFUS: magnetic resonance-guided high-intensity focused ultrasound
MRI: magnetic resonance imaging
RF: radiofrequency
RFA: radiofrequency ablation
ROI: region of interest
SSI: supersonic shear imaging
USgFUS: ultrasound-guided high-intensity focused ultrasound
